# DINI: data imputation using neural inversion for edge applications

**DOI:** 10.1038/s41598-022-24369-1

**Published:** 2022-11-23

**Authors:** Shikhar Tuli, Niraj K. Jha

**Affiliations:** Department of Electrical and Computer Engineering, Princeton, NJ 08540 USA

**Keywords:** Computer science, Electrical and electronic engineering

## Abstract

The edge computing paradigm has recently drawn significant attention from industry and academia. Due to the advantages in quality-of-service metrics, namely, latency, bandwidth, energy efficiency, privacy, and security, deploying artificial intelligence (AI) models at the network edge has attracted widespread interest. Edge-AI has seen applications in diverse domains that involve large amounts of data. However, poor dataset quality plagues this compute regime owing to numerous data corruption sources, including missing data. As such systems are increasingly being deployed in mission-critical applications, mitigating the effects of corrupted data becomes important. In this work, we propose a strategy based on data imputation using neural inversion, DINI. It trains a surrogate model and runs data imputation in an interleaved fashion. Unlike previous works, DINI is a model-agnostic framework applicable to diverse deep learning architectures. DINI outperforms state-of-the-art methods by at least 10.7% in average imputation error. Applying DINI to mission-critical applications can increase prediction accuracy to up to 99% (F1 score of 0.99), resulting in significant gains compared to baseline methods.

## Introduction

In the past decade, the Internet-of-Things (IoT) paradigm has seen an explosion in its adoption by businesses across continents and industries^1^. The number of IoT devices worldwide is forecast to almost triple from 9.7 billion in 2020 to more than 29 billion in 2030^2^. This burgeoning success has been made possible by the increasingly affordable and accessible low-power compute platforms. These platforms have fueled the growth of edge-AI, bringing computationally-expensive AI methods to the network edge^3–5^. A major driving force behind training/inference of deep neural network (DNN) models on the network edge is the advantages they provide in latency, bandwidth, energy efficiency, privacy, and security, relative to traditional cloud-based approaches^6^. The edge computing paradigm primarily requires collecting data from various sensors. Cyber-physical systems (CPS) also involve sending actuation signals to multiple devices in a physical environment. Other applications, where edge computing has made significant strides, include smart healthcare^7^, nuclear power plants^8^, smart grids^9^, and autonomous vehicles^10^, to name a few. However, corrupted sensor data or partially-procured/missing data plague these applications. Recently, DNN-based approaches have shown promise in effectively imputing missing data^11^. However, as we show in this work, even state-of-the-art DNN-based methods become ineffective when edge-specific corruptions are present (*e.g.*, where output labels may be missing even when all input feature values are available, or when some feature values may be missing). We propose a novel interleaved training-and-imputation approach, leveraging a DNN-based surrogate model to reliably impute the corrupted data (this includes missing data). We also propose unconventional methods to mimic data corruption, going beyond traditional techniques, to be more in accordance with corrupted data found in edge applications. We show that our imputation framework outperforms baseline methods on corrupted data synthesized through traditional and proposed corruption techniques.

### Challenges

Imputing corrupted/missing data is a challenging problem (we use the words *corrupted* and *missing* synonymously in this article; not-a-number, or NaN, values are often used to report missing data in the literature, and in the context of edge applications, we assume that which data are corrupted is known *a priori* through signal processing or other methods^11^). Missing data may be out-of-distribution relative to observed data, making it hard to predict the missing values^12^. This calls for generalizable models that can reliably impute the missing data. The imputation algorithm should be able to learn the underlying data-generation process (thus forming a surrogate model for this process) to effectively predict what data would be observed if they were not missing. Traditional methods typically implement interpolations on observed data^13,14^. Recent DNN-based approaches have shown substantial gains, but are restricted to either input feature imputation or output label prediction, limiting them to only specific scenarios^15,16^. In multi-input/multi-output regression datasets, it is possible that both the input and output features are corrupted, and thus only partially available. In this context, we need to impute not only the input but also the output features.

### Motivation

Corrupted data are commonplace in edge applications. Data can get corrupted in a variety of ways. In a distributed compute setting, network congestion can cause some data to reach late, resulting in some data becoming stale. Sensors may die due to a multitude of reasons—malfunctioning hardware, intermittent power supply, and even human-in-the-loop accidents^12^. Sensors and other edge devices are also prone to security attacks that may cause parts of the network to shut down or transmit malicious or corrupted data. Mission-critical edge deployments exacerbate this problem, where data corruption could hamper operation. Consider the following examples.

The first example is a chemical plant. There have been more than 50,000 reported hazardous chemical incidents in the last decade in the USA^17^. In chemical plants, where the formation of combustible gases is highly likely, it is important to quickly and reliably detect the appearance of such gases so that relevant action can be taken to alleviate their ill effects. For this application, we use the ‘Gas’ dataset^18^, which involves a mixture of different gases. The second example is a water distribution system that may be used in a nuclear power plant. As such facilities get smarter, it is important to quickly detect attacks on them to reduce the chances of large-scale calamities. The number of attacks on CPS is increasing by the day. Just in the first half of 2021, there were 1.5 billion IoT/CPS breaches reported^2^. These could adversely affect high-stakes organizations and facilities like nuclear plants. Thus, it is crucial to detect whether an attack has occurred so that corresponding mitigating mechanisms can be invoked. For this application, we use the ‘smart water treatment’ (SWaT) dataset^19^. Finally, Internet of Medical Things (IoMT) is a growing industry with a current market size of $42 billion. In applications like the smart detection of COVID^20^, some data may either be corrupted or simply unavailable. Even under these circumstances, it may be of interest to reliably detect disease onset in a secure and private (in terms of inference on the network edge) manner. Since data may be scarce in such critical applications, simply throwing away corrupted data may not be a viable option.

### Contributions

In this work, we aim to address the challenge of data imputation by proposing a DNN-based surrogate modeling approach—data imputation using neural inversion (DINI). We leverage gradient-based optimization using backpropagation to the input (GOBI)^21^, implemented through neural inversion^22^. DINI implements interleaved training (of the surrogate model) and imputation (of the data). As a surrogate model is trained, it can impute the corrupted data better, making an even superior model available for the next training iteration. We hypothesize that an interactive dynamic between imputation and training ensures more informed data generation and surrogate modeling. DINI can handle variegated data types, including multi-input/multi-output datasets. Input data can be continuous or categorical; the output may also have categorical labels or continuous values. Unlike previous works^15,16^, DINI can work with diverse types of DNN models, from fully-connected neural networks (FCNNs) to advanced architectures like Transformers^23^, whichever model works best for the given data distribution and model setting. Finally, DINI can output the uncertainty in predicted values like recent works^15^.

**Figure 1:**
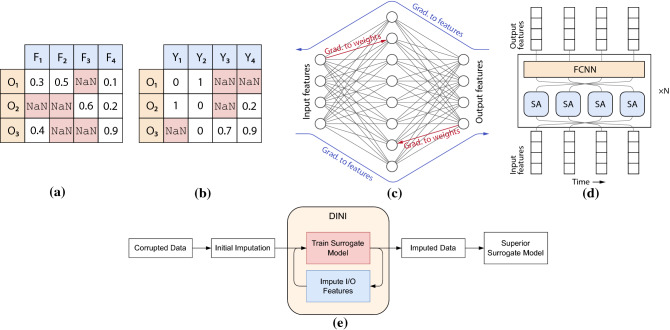
DNN-based data imputation and surrogate modeling framework of DINI. Example (**a**) input and (**b**) output tabular data. Supported surrogate models: (**c**) FCNN-based and (**d**) Transformer-based for time-series data. (**e**) High-level schematic of the DINI pipeline.

Figure 1 shows a high-level working schematic of the DINI framework. Tabular input (with features $\text {F}_1$–$\text {F}_4$) and output data (with features $\text {Y}_1$–$\text {Y}_4$) support both continuous and categorical features, along with their combinations. Figure 1a and b show these, respectively. We show only the first three observations (rows $\text {O}_1$–$\text {O}_3$). NaN values represent corrupted data. Output features $\text {Y}_1$ and $\text {Y}_2$ are categorical (may or may not be one-hot encoded). Previous works often refer categorical one-hot encoded output features as *output labels*. Since we support an expanded set of output formats, like the inputs, we refer to them as output features instead. Figure 1c shows DINI leveraging a DNN-based surrogate model (here, an FCNN) to map the input to the output and vice versa. During training, we backpropagate the gradients (from an appropriate loss function) to the weights (shown in red). During imputation, we freeze the model weights and backpropagate the gradients to the input/output features to predict the missing values (shown in blue). Figure 1d shows a Transformer-based surrogate model for time-series data, supported by DINI. Only one encoder layer is shown (it can be repeated *N* times) with four self-attention (SA) heads followed by an FCNN. Figure 1e shows a high-level schematic of the DINI pipeline. We first impute the corrupted data (with NaN values) with an initial imputation method (details in section “Methodology”) and then forward them to the DINI framework. DINI implements an interleaved training-and-imputation pipeline, which iteratively trains the surrogate model and imputes the data based on the updated model in a repeated fashion. This not only outputs an imputed dataset with no corruptions, but also a superior surrogate model that better represents the data distribution.

DINI outperforms baseline methods by at least 10.7% in reducing average error across diverse datasets. We further demonstrate the effectiveness of DINI in three case studies involving mission-critical edge applications. Moreover, we propose novel corruption techniques motivated by the distribution of corrupted data found in edge-AI settings. We show that DINI outperforms baseline approaches, giving much higher prediction performance for the required label.

### Outline

The rest of the article is organized as follows. Section “Background and related work” discusses background material on data corruption strategies, related works on imputation, and their critique. Section “Methodology” presents the DINI framework in detail. Section “Experimental setup” describes the experimental setup and presents the datasets used and baseline approaches for comparison. We validate our proposed framework and discuss the results in section “Results”. Section Discussion discusses limitations and future work directions. Finally, section Conclusions concludes the article.

## Background and related work

Various synthetic corruption methods have been widely used in the literature. We give a brief overview of these methods in this section. We then describe related works on data imputation and highlight their limitations.

### Synthetic corruption methods

As pointed out before, corrupted data are inherently assumed to be missing. Mathematically, let the data be denoted by a matrix-valued random variable $\mathbf {X} \in \mathbb {R}^{n \times d}$, where *n* is the number of observations (rows) and *d* is the data dimension (columns). Now, $\mathbf {x}$ denotes a realization of $\mathbf {X}$ and $\tilde{\mathbf {x}}$ denotes its observation. Note the difference between *realized* and *observed* values of the data^24^. The observed value is a function of the instantiation of the random variable for the data and its missingness. More concretely, let $\mathbf {M}$ denote the missingness in input data (it has the same dimensions as $\mathbf {X}$). The $(i, j)\mathrm{th}$ element of $\mathbf {M}$ is 1 if the corresponding element of $\mathbf {X}$ is observed and 0 if it is missing. In summary, $\mathbf {x} \sim \mathbf {X}$ and its observation is a function of $\mathbf {x}$ and $\mathbf {m}$, *i.e.*, $\tilde{\mathbf {x}} = o(\mathbf {x}, \mathbf {m})$, where $\mathbf {m} \sim \mathbf {M}$, such that:

$$\begin{aligned} \tilde{\mathbf {x}}_{ij} = {\left\{ \begin{array}{ll} \mathbf {x}_{ij}, &\text {if } \mathbf {m}_{ij} = 1\\ \texttt {NaN}, &\text {otherwise} \end{array}\right. } \end{aligned}$$

For the purpose of surrogate modeling, $\tilde{\mathbf {x}}$ is divided based on input and output feature columns as $\tilde{\mathbf {x}} = \left[ \tilde{\mathbf {x}}_{in} \ \tilde{\mathbf {x}}_{out}\right]$, where $[ \cdot ]$ denotes concatenation of matrices in block notation. Here, $\tilde{\mathbf {x}}_{in} \in \mathbb {R}^{n \times d_{in}}$ and $\tilde{\mathbf {x}}_{out} \in \mathbb {R}^{n \times d_{out}}$. The observed data can be further categorized into correctly observed (denoted by $\tilde{\mathbf {x}}^{o}$) or corrupted (denoted by $\tilde{\mathbf {x}}^{c}$) values. Table 1 summarizes the notations used in this work.

**Table 1:** Notations used in DINI.

Notation	Definition
*n*	The number of observations (rows in the tabular dataset)
*d*	The data dimension (columns in the tabular dataset)
$d_{in}$	The input data dimension; $d_{in} < d$
$d_{out}$	The output data dimension; $d_{out} < d$
$\mathbf{X}$	Matrix-valued random variable, $\mathbf {X} \in \mathbb {R}^{n \times d}$ for the data distribution
$\mathbf {x}$	A realization of $\mathbf {X}$, *i.e.*, $\mathbf {x}$ is sampled from the data distribution, $\mathbf {x} \sim \mathbf {X}$
$\mathbf {M}$	Matrix-valued random variable, $\mathbf {M} \in \mathbb {R}^{n \times d}$ denoting the distribution of missingness in the data
$\mathbf {m}$	A realization of $\mathbf {M}$, *i.e.*, $\mathbf {m}$ is sampled from the missingness distribution, $\mathbf {m} \sim \mathbf {M}$
$\mathbf {m}_{in}$	Part of the missingness matrix corresponding to the input; $\mathbf {m}_{in} \in \mathbb {R}^{n \times d_{in}}$
$\mathbf {m}_{out}$	Part of the missingness matrix corresponding to the output; $\mathbf {m}_{out} \in \mathbb {R}^{n \times d_{out}}$
$o(\cdot , \cdot )$	Observation function, given a realization and missingness matrix
$\tilde{\mathbf {x}}$	An observation of $\mathbf {x}$ that has missing NaN values; $\tilde{\mathbf {x}} = o(\mathbf {x}, \mathbf {m})$
$\tilde{\mathbf {x}}_{in}$	Part of the observed data corresponding to the input; $\tilde{\mathbf {x}}_{in} \in \mathbb {R}^{n \times d_{in}}$
$\tilde{\mathbf {x}}_{out}$	Part of the observed data corresponding to the output; $\tilde{\mathbf {x}}_{out} \in \mathbb {R}^{n \times d_{out}}$
$\tilde{\mathbf {x}}^{o}$	Correctly observed data, formed by the rows of $\tilde{\mathbf {x}}$ with no NaN values
$\tilde{\mathbf {x}}^{c}$	Corrupted observed data, formed by the rows of $\tilde{\mathbf {x}}$ with NaN values
$\phi$	Missingess model that generates the missingness distribution $\mathbf {M}$
$\hat{\mathbf {x}}$	Final imputed dataset; the imputation method takes in $\tilde{\mathbf {x}}$ and outputs $\hat{\mathbf {x}}$
$f_{\theta _1}$	Forward surrogate model (from input to output) with trainable weights $\theta _1$
$b_{\theta _2}$	Backward surrogate model (from output to input) with trainable weights $\theta _2$
$\mathcal {F}$	Overall surrogate model for input and output predictions, a combination of the above two: $\mathcal {F} = (f_{\theta _1}, b_{\theta _2})$
$\epsilon _*$	Convergence threshold for the corresponding operation $*$
$\eta _1$, $\eta _2$	Learning rates for updating weights for the forward and backward models
$\eta _{in}$, $\eta _{out}$	Learning rates for updating input and output features
$\mathcal {L}^f$, $\mathcal {L}^b$	Loss functions for the forward and backward models
$\nabla _*$	Gradient w.r.t. $*$; where $*$ could be model weights ($\theta _1$, $\theta _2$) or previously imputed features ($\hat{\mathbf {x}}_{in}$, $\hat{\mathbf {x}}_{out}$)

Here, the reader may notice a difference between our definition of *observed* values from those used in the literature^24^. Realized data are the data we would get when there is no source of corruption. Observed data are the complete data that we see *with* the corruption (*i.e.*, with NaN values). The part of the observed data that is correct, unlike previous works, is called *correctly observed* data ($\tilde{\mathbf {x}}^o$); part of the data that is corrupted/missing is simply called *corrupted* data ($\tilde{\mathbf {x}}^c$). The slight change in notation is motivated by the need to unify previous inconsistencies^12,15,24,25^ and bind our formulation to the context of data corruption.

Rubin^26^ has defined a widely used, yet controversial^24^, nomenclature for synthetic corruption (or missing value) mechanisms. We present these next.

#### Missing completely at random

The first is missing completely at random (MCAR). In MCAR, the data are corrupted entirely at random, *i.e.*, there is no dependency on the data. Consider a hypothesized missingness model $\phi$. Then, as per the MCAR scheme:

$$\begin{aligned} P_\phi (\mathbf {M}|\tilde{\mathbf {x}}^{o}, \tilde{\mathbf {x}}^{c}) = P_\phi (\mathbf {M}) \end{aligned}$$

In other words, the missing values do not depend on either the correctly observed or the corrupted values, which constitute the observed data $\tilde{\mathbf {x}}$. Here, $\phi$ is a uniform sampling model that corrupts data completely randomly.

#### Missing at random

The term missing at random (MAR) is a misnomer. Basically, MAR corruption refers to the missingness depending solely on the correctly observed data, or:

$$\begin{aligned} P_\phi (\mathbf {M}|\tilde{\mathbf {x}}^{o}, \tilde{\mathbf {x}}^{c}) = P_\phi (\mathbf {M}|\tilde{\mathbf {x}}^{o}) \end{aligned}$$

Here, $\phi$ is a logistic missingness model^25^. First, a subset of variables (columns) with *no* missing values is randomly selected. The remaining variables have missing values based on a logistic model with random weights, depending on the correctly observed data, rescaled to attain the desired proportion of missing values for those variables.

#### Missing not at random

Data are said to be missing not at random (MNAR) if the missingness is neither MCAR nor MAR. More specifically, data are MNAR if the missingness depends on the correctly observed and potentially even the corrupted values. In this context, the missingness cannot be fully accounted for by the correctly observed values. Here, we implement $\phi$ as a self-masking logistic model^25^. The values are masked based on a probability given by the logistic model with random weights, having the entire data matrix $\mathbf {x}$ as input.

#### Missing streams at random

To go beyond traditional corruption schemes, we propose two corruption techniques inspired by the distribution of corrupted data in diverse edge deployments^11,12^. Sensor data from various sources in a distributed IoT network can get corrupted, and once corrupted, likely stay corrupted for extended periods of time before being reset. To account for such scenarios, we propose the missing streams at random (MSAR) corruption technique. In this case, the missingness model $\phi$ chooses points in the data matrix at random and, unlike MCAR, corrupts a stream (of length 10 in our experiments) of datapoints through that column. This model is especially relevant to time-series data.

#### Missing patches at random

To account for spatiotemporal correlation in the corruption process, we further propose the missing patches at random (MPAR) corruption mechanism. In a distributed environment, sensors are often closely placed in groups (to implement redundancy in some cases). For example, some sensors might be placed in one part of a smart facility and others in another. If one sensor fails in a group, several sensors in the group may likely fail. Thus, rather than streams (involving a single column), patches of data will get corrupted. Here, $\phi$ chooses points in the data matrix randomly, then corrupts a patch (of size $5 \times 5$ in our experiments) around that point.

### Data imputation methods

We can categorize previously proposed imputation methods as either discriminative or generative. Discriminative methods include multivariate imputation by chained equations (MICE)^14^, matrix completion^27^, spectral regularization^28^, iterative singular value decomposition (SVD)^29^, and *k*-nearest neighbors (kNN)^13^. Generative models include algorithms based on expectation maximization, such as those using Gaussian mixture models (GMMs)^30^ and approaches based on modern deep learning, like denoising autoencoders (DAEs)^31,32^ and generative adversarial networks (GANs). One state-of-the-art GAN-based imputation method is GAIN^15^, which forgoes the assumptions made in previous generative imputation models—restrictions on the underlying data distribution and types of datasets (categorical or continuous). GRAPE^16^ is yet another DNN-based approach that converts the data into a bipartite graph and then uses a graph neural network (GNN) for imputation.

Traditional statistical methods for imputation provide useful theoretical bounds but exhibit notable shortcomings. First, they tend to make strong assumptions about the data distribution. Second, they lack flexibility for handling mixed data types that include both continuous and categorical variables. Finally, matrix-completion-based approaches do not generalize to unseen samples (thus performing poorly on out-of-distribution data) and require retraining when new data samples are encountered^13,27–29^. Recent DNN-based approaches try to address these shortcomings but are still limited in their application. GAIN only implements input feature imputation and assumes that all output labels are available^15^. GRAPE does either input feature imputation or output label prediction, but not both^16^. It also does not support uncertainties in prediction, only models the *expectation* of the data distribution. Other recent works that use these methods, or their combination, are only applicable to specific applications^33^. In many applications, especially in the context of edge deployments, both input features and output labels may be missing^12^. Further, the output features in previous works are only one-dimensional (only one continuous feature or categorical label). These restrictions prevent their application to many tasks, including multi-input/multi-output regression datasets. In the case of such datasets and under some corruption strategies (e.g., when the output can also be corrupted), even state-of-the-art DNN-based approaches become ineffective, as we demonstrate later. DINI, on the other hand, can support mixed continuous and categorical features not only in the input but also in the output. Lastly, previous DNN-based works are either restricted to adversarial networks^15^, autoencoders^31^, or GNNs^16^. However, different DNN models may be suitable for different data distributions. DINI, being a model-agnostic framework, can be applied to diverse DNN architectures, including FCNNs, convolutional neural networks (CNNs)^34^, long-short term memories (LSTMs)^35^, and even Transformers^23^.

## Methodology

We now discuss the DINI framework in detail.

### Problem formulation

As noted previously, we consider the imputation (via surrogate modeling) of the observed dataset $\tilde{\mathbf {x}} \in \mathbb {R}^{n \times d}$, partitioned into input and output columns as $\tilde{\mathbf {x}}_{in} \in \mathbb {R}^{n \times d_{in}}$ and $\tilde{\mathbf {x}}_{out} \in \mathbb {R}^{n \times d_{out}}$. For better-posed modeling, we first scale the input data to [0, 1] with a MinMax scaler^36^. The task at hand is to output an imputed dataset $\hat{\mathbf {x}}$ that is as close as possible to the real dataset $\mathbf {x}$, had there not been any corruption. The goal is to achieve the least error between the imputed and real data. The two error metrics are the root mean square error (RMSE) and mean absolute error (MAE)^37^, defined as follows:

$$\begin{aligned} \text {RMSE}(\mathbf {x}, \hat{\mathbf {x}})&= \sqrt{\frac{1}{nd} \sum _{ij} \left( x_{ij} - \hat{x}_{ij} \right) ^2}, \quad \forall \ x_{ij} \in \mathbf {x}, \ \hat{x}_{ij} \in \hat{\mathbf {x}}\\ \text {MAE}(\mathbf {x}, \hat{\mathbf {x}})&= \frac{1}{nd} \sum _{ij} | x_{ij} - \hat{x}_{ij} |, \quad \forall \ x_{ij} \in \mathbf {x}, \ \hat{x}_{ij} \in \hat{\mathbf {x}} \end{aligned}$$

Note that from the $\texttt {NaN}$ values in $\tilde{\mathbf {x}}$, the missingness mask $\mathbf {m} \in \mathbb {R}^{n \times d}$ is recoverable and can also be similarly partitioned into $\mathbf {m}_{in} \in \mathbb {R}^{n \times d_{in}}$ and $\mathbf {m}_{out} \in \mathbb {R}^{n \times d_{out}}$.

### The DINI framework

DINI comprises two DNNs that act as surrogate models for the data distribution. Each DNN models one side (input-to-output or output-to-input) of the dataset and runs GOBI for imputation. Thus, the surrogate model of DINI is given by $\mathcal {F}$ that comprises two functions, one being the forward model $f_{\theta _1}: [0, 1]^{d_{in}} \rightarrow [0, 1]^{d_{out}}$ and the other the backward model $b_{\theta _2}: [0, 1]^{d_{out}} \rightarrow [0, 1]^{d_{in}}$. Here, $\theta _1$ and $\theta _2$ are the parameters, or weights of the DNNs, for the forward and backward models, respectively. DINI involves interleaved training of the surrogate model $\mathcal {F}$ (where the neural network parameters $\theta _1$ and $\theta _2$ are updated) and imputation (where the $\hat{\mathbf {x}}$ data are updated).

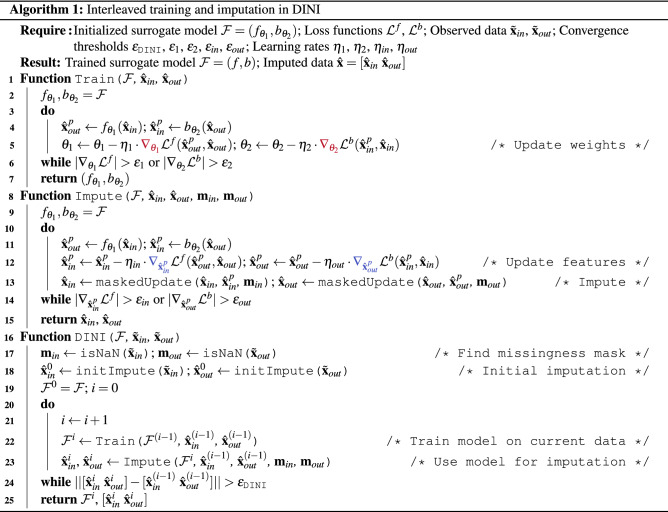



Algorithm 1 summarizes this interleaved training-and-imputation pipeline. First, isNaN () recovers the missingness masks in the input and output data (line 17). Then, initImpute () takes the observed data and outputs them after running an initial imputation on the $\texttt {NaN}$ values so that the data are amenable to training the surrogate model (line 18). This could be either mean, random, or zero imputation. Based on our tests, zero imputation performs the best. This could be attributed to the high gradient of the logistic function at zero, leading to faster convergence for the corrupted values. Then, we run interleaved training and imputation until convergence (lines 22-23). Here, when the new imputed data gets close enough to the old data based on a threshold $\epsilon _{\texttt {DINI}}$ (line 24), the algorithm reaches convergence. During training, the forward and backward models are trained by backpropagating the gradients of an appropriate loss function to their respective parameters ($\theta _1$ and $\theta _2$; line 5). The red color shows the operation of gradients towards the weights. Here, we show stochastic gradient descent for simplicity, although we used the Adam optimizer^38^ in our experiments. To account for both continuous and categorical values in the input and output features, we consider the loss function as a sum of the RMSE and the MAE between the predicted and actual data matrices. Mathematically,

$$\begin{aligned} \mathcal {L}^f(\mathbf {x}, \hat{\mathbf {x}}) = \mathcal {L}^b(\mathbf {x}, \hat{\mathbf {x}}) = \text {RMSE}(\mathbf {x}, \hat{\mathbf {x}}) + \text {MAE}(\mathbf {x}, \hat{\mathbf {x}}) \end{aligned}$$

The loss function could also have leveraged the categorical cross-entropy loss, where the variables are known to be categorical and one-hot encoded. During imputation, the model weights are frozen and gradients are computed towards the respective inputs, *i.e.*, $\hat{\mathbf {x}}_{in}^p$ and $\hat{\mathbf {x}}_{out}^p$ (line 12). Again, blue type color represents the operation for gradients towards the features. We only impute that part of the data that is known to be corrupted, using the masks $\mathbf {m}_{in}$ and $\mathbf {m}_{out}$. Leveraging Monte Carlo (MC) dropout^39^, the forward and backward models output the data distribution, whose standard deviation gives the uncertainty. Partial imputation can be performed based on the least uncertain predictions. This is implemented by the maskedUpdate () function (line 13). If some variables are categorical, this function also forces the corresponding imputed values to 0 or 1 based on a threshold (set to 0.5). Training or imputation reaches convergence when the $L_1$-norm of the respective gradients falls below a threshold. Finally, the DINI () function outputs the trained surrogate model $\mathcal {F}$ along with the imputed data matrix $\hat{\mathbf {x}}$ (line 25). Note that, unlike what Figure 1c shows, we implement the surrogate model as a set of two functions ($f_{\theta _1}$ and $b_{\theta _2}$) that we train in tandem. This aids the implementation of GOBI in a conserved manner. We defer the implementation of DINI using weight-shared models, or even a single model, to future work.

## Experimental setup

In this section, we discuss details of the experimental setup. First, we present the model architecture and training hyperparameters. We then discuss the datasets used for the imputation problem and the surrogate modeling tasks for three mission-critical edge applications. Finally, we briefly discuss the baselines used for comparison with the DINI model.

### The model architecture

As explained in section “The DINI framework”, we implemented the forward and backward models as two DNNs. For our experiments, we chose the DNNs to be FCNNs with the input and output number of neurons equal to the corresponding data dimensions. More concretely, for the forward model *f* (backward model *b*), we set the number of input neurons to $d_{in}$ ($d_{out}$) and the number of output neurons to $d_{out}$ ($d_{in}$). We ran a grid search over the number of hidden layers and the dimension of each hidden layer. We found that the smallest architecture that achieved a reasonable RMSE ($<1 \times 10^{-3}$) on the *uncorrupted* data (for all considered datasets) needs only one hidden layer with 512 neurons. We use leaky ReLU as the activation function for each layer except for the output layer, where we use the sigmoid activation function. Any DNN-based surrogate model can leverage DINI. Hence, for time-series datasets, we further tested LSTM-based^35^ architectures and Transformers^23^ as well. Figure 1d shows how a Transformer-based surrogate model employs DINI. However, we found that for the datasets considered, FCNNs were the simplest architectures that also performed the best in imputation performance (see section “Ablation analysis”). We leave other applications with more complex data distributions that require DINI with advanced deep learning models for future exploration. We set the hyperparameters for the DINI pipeline as follows. We set the learning rates to $\eta _1 = \eta _2 = 1 \times 10^{-4}$, $\eta _{in} = \eta _{out} = 5 \times 10^{-4}$. We use a weight decay of $1 \times 10^{-3}$. We set Adam optimizer’s parameters to $\beta _1 = 0.9$, $\beta _2 = 0.999$. Finally, we set all convergence thresholds to $1 \times 10^{-3}$.

### Imputation datasets

To measure the imputation performance (in terms of RMSE and MAE), we consider a diverse set of popular machine learning datasets, including those used by previous works^15,16^. These datasets include ones from the popular UCI repository^40^: breast cancer Wisconsin prognostic dataset (Breast), energy efficiency dataset (Energy), and the yacht hydrodynamics dataset (Yacht). Since DINI can also tackle multi-output datasets, we consider such datasets as well. For this, we consider two prediction outputs in the Energy dataset: separate heating and cooling loads, which previous works do not^16^. We also consider other popular datasets like the Diabetes dataset^41^ (with six blood serum estimates and the responses of interest as continuous-valued outputs), the Diamonds dataset^42^ (with carat and price as two continuous-valued prediction outputs), and the Flights dataset^43^ (with two categorical outputs, namely whether the flight was diverted or canceled, and three continuous outputs: departure and arrival delays along with the estimated flying time). Further, unlike previous works, we carry out corruption not only on input features but also on the output features.

### Case studies

For case studies related to mission-critical edge applications, we consider three datasets, as described in section “Motivation”. The first is the Gas dataset^18^ that is from the UCI repository^40^. It contains mixtures of gases at different concentrations. In the context of detecting flammable gases, we take measurements from 15 sensors as input and set the detection label for flammable gases as the categorical output. The second is the SWaT dataset^19^ with a diverse set of categorical and continuous input features, and detection of attack as the prediction label. Finally, we consider the smart-COVID detection dataset^20^ that considers age, sex, offset of days since symptoms appeared, type of pneumonia, and features extracted from chest X-rays^44^.

### Baselines

To validate DINI’s imputation and surrogate modeling performance, we compare it against various baselines, as mentioned in section “Data imputation methods”. For completeness, we present these commonly used imputation methods below:

- Mean/median imputation: The method imputes the corrupted values $\tilde{\mathbf {x}}_{ij}$ with the mean/median of all correctly observed samples along column *j*.
- kNN imputation: The method imputes the corrupted rows *i* in $\tilde{\mathbf {x}}_{ij}$ based on the kNN along column *j* with the weights based on the Euclidean distance to the row.
- SVD imputation: The method imputes missing values based on matrix completion with iterative low-rank SVD decomposition.
- MICE imputation: The method runs multiple regressions where each missing value is modeled conditioned on the observed non-missing values.
- Spectral imputation: This matrix completion model uses the nuclear norm as a regularizer and imputes missing values with iterative soft-thresholded SVD.
- Matrix imputation: This method finds the matrix with the minimum nuclear norm that fits the correctly observed data.
- GMM imputation: This approach fits a GMM on the observed data using the expectation-maximization algorithm and imputes the missing values based on the model.
- GAIN imputation: A generative-adversarial-network-based input feature imputation strategy.
- GRAPE imputation: A state-of-the-art imputation method that converts data into a bipartite graph and uses a GNN model for imputation.

GAIN only does input feature imputation. GRAPE either implements input feature imputation or output label prediction, but not both simultaneously. We adapt these models, based on the new formulation of DINI, as a forward and a backward model. We then apply these methods to the input and output features based on these models. We call these adaptations GAIN$^*$ and GRAPE$^*$.

The time complexity of the proposed DINI algorithm (see Algorithm 1) is $\mathcal {O}(n d^2)$ for one iteration of imputation of the entire dataset. This is because the forward pass of an FCNN (and even backpropagation) implements matrix multiplication operations in practice. For the considered architecture ($d_{in} < d$ input neurons, 512 hidden neurons, $d_{out} < d$ output neurons), this is implemented in $\mathcal {O}(n d^2)$ time. The same is true for both training the surrogate model and imputation. Here, training and imputation are assumed to be for a fixed number of epochs. Classical approaches like Mean and Median have $\mathcal {O}(nd)$ time complexity. kNN has a time complexity of $\mathcal {O}(knd)$. On the other hand, state-of-the-art DNN-based methods, GAIN and GRAPE, have time complexities $\mathcal {O}(nd^2)$ and $\mathcal {O}(rnh^2)$, respectively, where *r* is the number of neighbors sampled for each node and *h* is the node hidden feature dimension^45^. The number of hidden layers is assumed to be one for both these methods. This implies that DINI is comparable to previous DNN-based methods in computational complexity.

## Results

This section presents performance comparisons for DINI with baseline imputation methods. Since DINI inherently works with a DNN-based surrogate model, we subsequently present its modeling performance by testing the corresponding label detection performance on three mission-critical edge applications. Finally, we present ablation studies.

### Imputation performance

We compare DINI with the baseline imputation methods described in section “Baselines”. For this comparison, we test the RMSE and MAE of the imputed data relative to the actual data when subjected to different corruption strategies (including the two newly proposed ones). Table 2 compares the imputation performance of DINI across six datasets and five corruption strategies against the considered baselines. DINI outperforms the baselines for most tasks (46 out of 60 rows). Spectral imputation performs the worst on most datasets. GAIN$^*$ does not perform well on the Yacht dataset when subjected to corruption in both the input and output features. On an average, DINI outperforms the next best imputation method, i.e., MICE, by 10.7% in terms of imputation error. Even though MICE inherently assumes the corruption to be either MCAR or MAR, DINI achieves a lower error even under these strategies for most datasets. Unlike the results presented in previous works^15,16^, as we see here, even state-of-the-art DNN-based methods are not that effective when subjected to simultaneous input/output corruption. DINI outperforms GAIN$^*$ and GRAPE$^*$ by 36.8% and 33.9%, respectively.

**Table 2:**
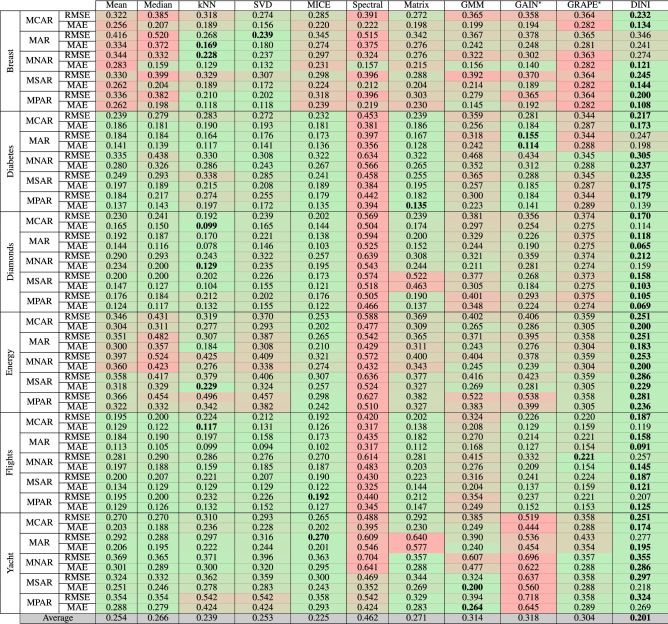
Comparison of imputation performance of DINI with various baselines.

Six datasets and five (including two newly proposed) corruption strategies are considered. The last row averages all entries for each column (including both RMSE and MAE values). Confidence intervals are not shown to conserve space.

Values corresponding to the lowest error are in [bold].

### Surrogate modeling performance

**Figure 2:**
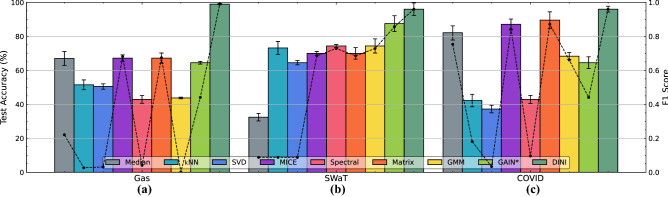
Test accuracy and F1 score for imputation models on the (**a**) Gas, (**b**) SWaT, and (**c**) COVID datasets, for the MSAR corruption strategy. Test accuracy is shown as bar plots with the axis on the left. F1 score is shown as a dashed line plot with the axis on the right.

**Figure 3:**
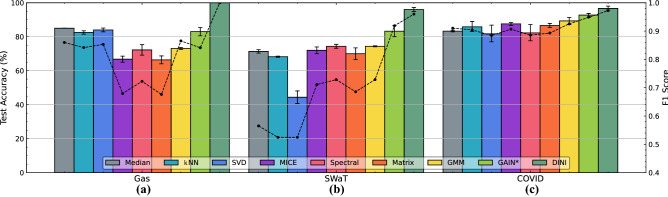
Test accuracy and F1 score for imputation models on the (**a**) Gas, (**b**) SWaT, and (**c**) COVID datasets, for the MPAR corruption strategy.

Since DINI is more than an imputation method, we can leverage the implicit surrogate training for tasks beyond filling missing values. Previous works have widely used surrogate training and inference; however, seamless exploitation of corrupted data (using interleaved imputation) is novel and is broadly applicable to edge applications where corrupted sensor data are commonplace. Hence, we leverage this extra capability of DINI to obtain better surrogate models for such applications. We use three mission-critical applications as case studies. We formulate the comparison experiments as follows. For each dataset, we split the data three ways: 40%-40%-20%. We assume 40% of the data is heavily corrupted (no row can be extracted that does not have any corrupted values). For this, we use MSAR or MPAR corruption with close to 100% corruption ratio. The first 40% of the uncorrupted data and the 40% corrupted data comprise the 80% training set for imputation and surrogate model training. We use the final 20% of the data as the test set. For like-to-like comparisons, with each imputation strategy, we use the same architecture for the surrogate model trained on the *imputed* data: FCNN with one hidden layer having 512 hidden neurons. Figures 2 and 3 show the modeling performance on the three datasets for imputed data from DINI and all the baseline methods. Note that we do not consider Mean imputation because it imputes categorical columns with an intermediate value that is not allowed (if the mean value is forced to 0 or 1 based on a threshold, the performance becomes close to that of Median imputation). GRAPE$^*$ is also not considered in these comparisons since it only outputs RMSE/MAE in imputations in its graph format and does not convert the imputed data back to the tabular format for surrogate modeling. For the Gas dataset, we need to detect whether the flammable gas is observed or not. For the smart water plant (SWaT dataset), we need to detect if the system has been attacked. On the other hand, for smart-COVID detection, we need to detect if the patient has the disease. Since all these datasets have a single categorical output, we train the forward model in DINI with binary cross-entropy loss. In all these tasks, we not only wish to leverage the corrupted, partially observed data, but also need a high true positive rate since false negatives would incur high risks in such applications. On the other hand, we also need a low false positive rate since invoking mitigating mechanisms could be costly, and performing them needlessly could result in large system overheads. Hence, we plot the F1 score along with the test accuracy.

DINI consistently outperforms the baseline imputation methods with a high test accuracy and F1 score. For example, DINI attains around 99% test accuracy and 0.99 F1 score on the Gas dataset, implying that almost all cases where a flammable gas is present are correctly detected. No other imputation strategy approaches this performance. For the SWaT and COVID datasets, DINI reaches around 96% (0.95) and 97% (0.96) average test accuracy (F1 score) across the two corruption strategies, respectively. However, for some imputation strategies, like Median imputation with the Gas dataset under MSAR corruption, the F1 score is very low even when the test accuracy is reasonable. This is because the surrogate model is heavily biased toward negative labels (since the model has not generalized well), having a high number of true negatives but few true positives. This results in a low F1 score. DINI does not suffer from this problem.

**Figure 4:**
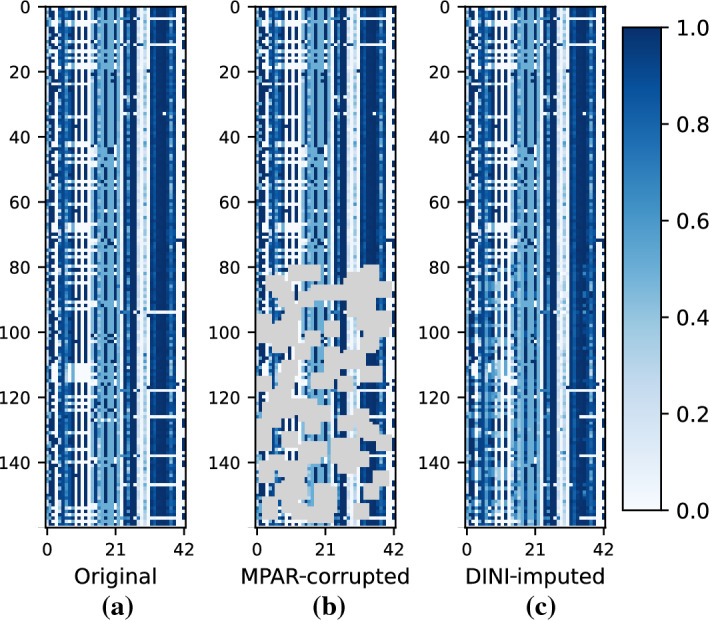
Data snapshot with only 160 rows from the SWaT dataset: (**a**) original, (**b**) MPAR-corrupted, and (**c**) DINI-imputed. 50% correct (0–79) and 50% corrupted (80–159; with a high corruption ratio) data form the training set for the imputation methods.

**Figure 5:**
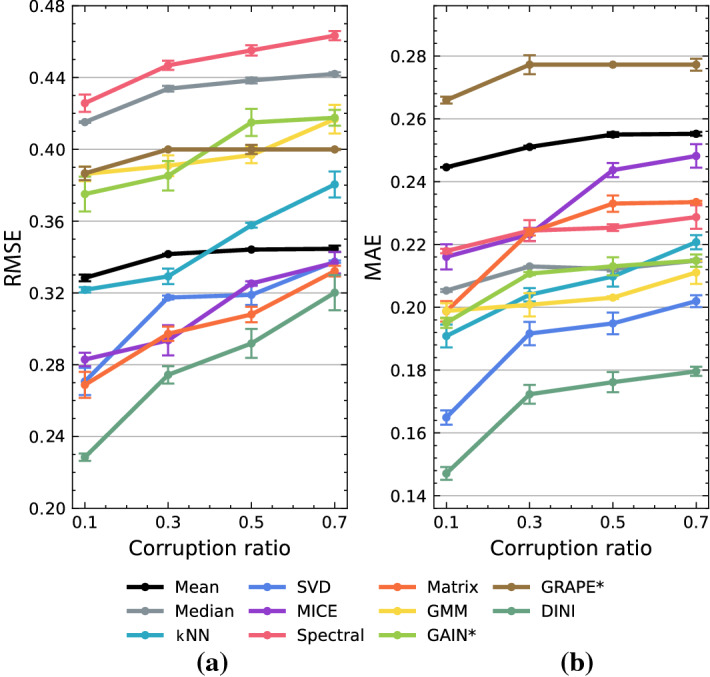
Error vs. corruption ratio for various imputation methods: (**a**) RMSE and (**b**) MAE. MCAR corruption on the Breast dataset was used for comparison.

Figure 4 shows how we passed the corrupted data to the imputation models in their training set. We observe that the data imputed by DINI, shown in Fig. 4c, are very similar to the original data, shown in Fig. 4a. This striking similarity shows that DINI can reproduce the underlying data distribution even in the presence of high levels of corruption. Figure 5 compares the imputation methods under different corruption ratios and the MCAR corruption strategy on the Breast dataset. DINI consistently outperforms baselines by achieving a lower RMSE and MAE for the different corruption ratios.

### Ablation analysis

**Table 3:** Ablation analysis for DINI.

Training and imputation method	RMSE	MAE
Pre-training on correct subset w/ imputation	0.412 ± 0.019	0.291 ± 0.015
Pre-training on correct subset w/ interleaved training/imputation	0.257 ± 0.029	0.163 ± 0.008
Interleaved training from scratch + partial imputation starting at 25$^{th}$ percentile	0.268 ± 0.030	0.169 ± 0.012
Interleaved training from scratch + partial imputation starting at 50$^{th}$ percentile	0.255 ± 0.024	0.160 ± 0.029
Interleaved training from scratch + partial imputation starting at 75$^{th}$ percentile	0.247 ± 0.019	0.151 ± 0.023
Interleaved training from scratch + complete imputation	**0.232** ± **0.019**	0.134 ± 0.013
w/ second-order gradients	0.233 ± 0.012	**0.133** ± **0.011**

Breast dataset was considered with MCAR corruption. Data is reported with 95% confidence intervals.

Values corresponding to the lowest error are in [bold].

To test the efficacy of our interleaved training-and-imputation strategy, we modify the DINI framework as follows. First, we train the surrogate model on the correctly observed subset and use this model for imputation (using GOBI for the input and output features from the forward and backward models). Second, we pre-train the surrogate model on the correct subset and run interleaved training and imputation on the corrupted subset. Note that we impute all the data at every iteration. Third, we run interleaved training and imputation from scratch (i.e., with no pre-training) on the entire dataset, as described in the DINI pipeline above. However, we attempt to leverage the uncertainty in prediction through the MC dropout layer. We thus only impute part of the data, where the model is the least uncertain. Based on the uncertainty values for the entire data matrix, we start at the 25th, 50th, or the 75th percentile of the uncertainties and impute only part of the data accordingly. To account for the surrogate model getting better towards the end of training, we linearly increase the imputation ratio to 100%. Table 3 shows the results on the Breast dataset with MCAR corruption (other datasets showed similar results). We observe that the method involving interleaved training and (complete) imputation from scratch outperforms previous approaches. Here, by complete imputation, we mean that 100% of the data are imputed at every iteration, regardless of the uncertainties. We explain this as follows. In the first approach, we do not leverage imputed data to improve the surrogate model further. In the second approach, after pre-training the surrogate model, training on the imputed data causes a *distribution shift*, as the model cannot train along with the correctly observed data. Finally, partial imputation adds to the bias present in the surrogate model initially, resulting in a higher imputation error. However, certain tasks requiring multiple solutions could benefit from the uncertainties in predictions. Taking inspiration from some recent works^46^ that leverage GOBI, we also tested second-order gradients using the AdaHessian optimizer^38^ in DINI’s surrogate model. This only provides marginal gains (reduction in MAE by 0.001) that are not statistically significant. Due to the high overhead of calculating these gradients, we stayed with first-order gradients in our experiments.

**Table 4:** Comparison of DNN-based models for DINI.

		LSTM (L-1, H-512, U)	LSTM (L-2, H-512, B)	LSTM (L-3, H-1024, B)	Transformer (L-1, H-512, A-4)	Transformer (L-3, H-512, A-8)	Transformer (L-6, H-1024, A-16)	FCNN (L-1, H-512)
Energy	Model Params.	49.7K	99.3K	297.9K	6.31M	18.92M	151.17M	**10.2K**
	RMSE	0.346 ± 0.022	0.302 ± 0.019	0.295 ± 0.030	0.351 ± 0.023	0.290 ± 0.019	0.283 ± 0.017	**0.281** ± **0.010**
	MAE	0.287 ± 0.018	0.255 ± 0.020	0.248 ± 0.031	0.287 ± 0.021	0.245 ± 0.012	0.242 ± 0.014	**0.236** ± **0.027**
Gas	Model Params.	81.9K	163.8K	491.5K	6.32M	18.93M	151.19M	**17.4K**
	RMSE	0.268 ± 0.021	0.253 ± 0.019	0.249 ± 0.025	0.298 ± 0.012	0.257 ± 0.018	0.251 ± 0.015	**0.248** ± **0.022**
	MAE	0.208 ± 0.012	0.202 ± 0.015	0.197 ± 0.015	0.222 ± 0.018	0.201 ± 0.012	0.197 ± 0.023	**0.193** ± **0.011**
SWaT	Model Params.	210.9K	421.9K	1.26M	6.35M	18.96M	151.24M	**46.1K**
	RMSE	0.505 ± 0.029	0.479 ± 0.037	0.473 ± 0.020	0.484 ± 0.026	0.467 ± 0.032	0.464 ± 0.027	**0.462** ± **0.018**
	MAE	0.421 ± 0.018	0.408 ± 0.016	0.392 ± 0.009	0.413 ± 0.022	0.393 ± 0.011	**0.385** ± **0.024**	0.387 ± 0.036

MPAR corruption was considered since it is the hardest to model in time-series datasets. ‘L’: number of layers (stacks in LSTM, hidden layers in FCNN), ‘H’: hidden dimension (number of neurons in the hidden layer for the FCNN), ‘U/B’: uni-/bi-directional model, ‘A’: number of attention heads in the Transformer. Values corresponding to the lowest number of model parameters or error are in [bold].

DINI supports diverse DNN-based surrogate models, including advanced architectures like LSTMs and Transformers. Table 4 compares these architectures with the FCNN used in our experiments for time-series datasets. FCNN performs slightly better than a Transformer with six encoder layers in most cases while being 24,603$\times$ smaller on average. This could be due to the FCNN having enough capacity for the chosen datasets, while the Transformer overfits on the training data resulting in lower performance.

## Discussion

As discused in section “Results”, DINI outperforms baseline methods in various experimental settings. The interleaved training-and-imputation pipeline enables high gains compared to the state-of-the-art methods. Further, it directly incorporates heterogeneous input and output feature formats (continuous, categorical, or a combination thereof). These advancements make it better at imputing data compared to traditional approaches. Unlike previous works, it is a unified framework that supports diverse DNN-based model architectures.

However, DINI has several limitations. For instance, it only imputes data that are *known* to be corrupted. One could also encounter adversarial data with fraudulent input feature values and noisy labeled data, where the corrupted data are not in the NaN form. Detecting such data falls under the scope of adversarial attack detection^47^ and confident learning^48^, respectively. One could extend the DINI model by incorporating aleatoric loss^49^ to account for such corruptions. We can also prune or correct the input or output entries with high uncertainties^50^ (after conversion to NaN values and subsequent imputation). We defer this to future work.

## Conclusions

In this article, we presented DINI, a pipeline for interleaved training of a surrogate model and imputation of data, leveraging gradients towards the input and output features in the model. DINI tackles corruption in both the input and output values, along with mixed continuous and categorical features in either. For better-posed problem formulation in edge-AI settings, we proposed novel corruption strategies that model the distribution of corrupted data in such applications more closely. We showed that DINI outperforms all baseline imputation methods, including state-of-the-art DNN-based models, achieving 10.7% lower imputation error relative to the next best baseline. Finally, we tested the modeling performance of DINI on mission-critical edge applications and showed that it can reach up to 99% test accuracy and 0.99 F1 score when detecting labels in such settings.

## Data Availability

All data and code are available in the supplementary files. The code and relevant testing scripts are made publicly available on GitHub under the BSD-3 license at https://github.com/jha-lab/dini.
